# A New Normal: An Age–Period–Cohort Analysis of the Transformed Childhood Adversity Landscape in the United States

**DOI:** 10.3390/bs16091687

**Published:** 2026-09-19

**Authors:** David Brown, Robert F. Anda, Laura E. Porter

**Affiliations:** 1 BCGI LLC/Pivot-23.5°, Cornelius, NC 28031, USA; 2 Developmental Adversity, Resilience, and Transformation Lab, School of Public and Community Medicine, University of Montana, Missoula, MT 59812, USA; 3 ACE Interface LLC, Peachtree City, GA 30269, USA; robanda@bellsouth.net; 4 ACE Interface LLC, Shelton, WA 98584, USA; lauraportergarden@gmail.com

**Keywords:** adverse childhood experiences, cohort effect, period effect, population surveillance, behavioral risk factor surveillance system

## Abstract

Adverse Childhood Experiences (ACEs) are strongly associated with poor physical, mental, and social outcomes, yet few studies have examined ACE prevalence across birth cohorts. We pooled Behavioral Risk Factor Surveillance System (BRFSS) data from 2009–2012 and 2019–2024 (1925–2003 birth cohorts) and analyzed them using an Age–Period–Cohort (APC) model. Early cohorts (circa 1930) reported a mean ACE score < 1 (prevalence of ACE score ≥ 4: ~1 in 20), whereas late 1990s/early 2000s cohorts reported a mean score > 2 (prevalence of ACE score ≥ 4: ~1 in 4). In APC models, the 1955–1979 cohorts had ~50% higher mean ACE scores (incidence rate ratio [IRR]: ~1.50) than pre-1930 cohorts. Additionally, ACE reporting was ~25% lower in 2009 than in 2019 (IRR = 0.75), indicating a strong period effect toward greater disclosure. At the population level, childhood adversity has shifted to a high-prevalence reality, likely reflecting both historical increases across cohorts and a shift toward greater disclosure over time. Addressing this cumulative burden requires integrated, intersectoral policy and safety-net reforms across the health, education, social services, and justice sectors.

## 1. Introduction

Adverse Childhood Experiences (ACEs) are traumatic events that occur before the age of 18 years—such as abuse, neglect, and household dysfunction (operationalized in this paper as *household adversities*)—and can disrupt neurological development and trigger chronic stress responses through epigenetic and physiological mechanisms (Felitti et al., 1998; Teicher & Samson, 2016; Yehuda & Lehrner, 2018; McKenna et al., 2023; Zhou & Ryan, 2023). These experiences are often summarized using a cumulative ACE score, where higher totals represent greater ACE exposure and are associated with a well-documented risk for chronic disease, mental health and substance abuse disorders, and premature mortality (Felitti et al., 1998; Anda et al., 2006; Brown et al., 2009; Yu et al., 2022). Because ACEs frequently co-occur, the ACE score serves as a powerful, albeit imperfect (Anda et al., 2020), proxy for the total burden of childhood adversity and its lifelong impact on health and social outcomes (Dong et al., 2004; Hughes et al., 2017).

**Figure 1:**
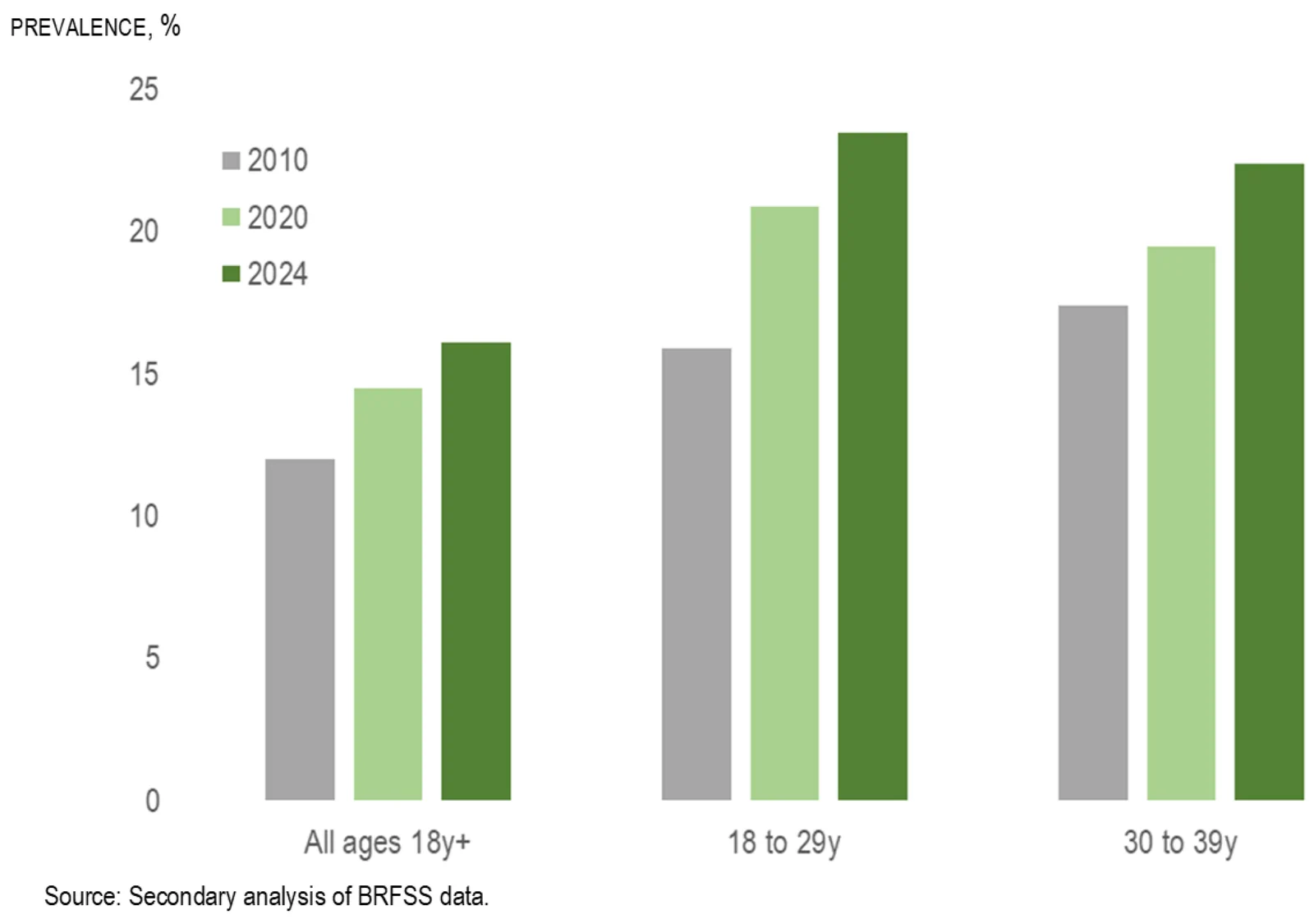
Prevalence of ACE scores ≥4 among all adults, and adults aged 18 to 29 years and 30 to 39 years from the 2010, 2020, and 2024 BRFSS data.

As the research on ACEs continues to grow (Struck et al., 2021), our understanding of their profound and lasting effects on the physical, mental, and social well-being of individuals, families, and communities also advances, with implications spanning generations (Schickedanz et al., 2021). Despite this growing evidence base, substantial gaps in our understanding remain, including in our understanding of the trends and patterns of ACE occurrence. In the original ACE Study, Felitti et al. (1998) observed a prevalence of ACE scores ≥4 of roughly 12.5% among mostly white, middle-class adults. Subsequent analysis of Behavioral Risk Factor Surveillance System (BRFSS) data on ACEs suggests recent increases in reported childhood adversity, particularly among young adults. For example, Figure 1 shows the estimated crude prevalence of ACE scores ≥4 among U.S. adults, which increased between 2010 and 2024 overall (in 2010: 12.0%, 95% CI: 11.1, 13.1; in 2020: 14.5%, 95% CI: 14.0, 15.0; in 2024: 16.1%, 95% CI: 15.2, 16.9) and within young and parenting-age adults (e.g., 30–39 years). In fact, we observe a +7.6 percentage-point increase for those aged 18 to 29 years (~48% relative increase), a +5.0 percentage-point increase for those aged 30 to 39 years (~29% relative increase), and a +4.1 percentage-point rise from 2010 to 2024 (or ~34% relative increase vs. 2010) overall. However, it is unclear whether these increases indicate an increase in the prevalence of childhood adversity or are reflective of other factors.

Here, using reported ACE data from adults aged 18 years or older collected via the BRFSS during two survey time periods, 2009–2012 and 2019–2024, we examine the historical occurrence of ACEs across successive birth cohorts to analyze the effects of contemporary changes in reporting (i.e., a person in 2024 is more likely to identify an event as “abuse” than a person in 1960, even if the event was the same). Central to this examination is the necessity of addressing “healthy survivor bias” and ACE-related morbidity on survey measures of their occurrence. Specifically, individuals with high levels of adversity face elevated risks of premature mortality (Brown et al., 2009; Yu et al., 2022) and institutionalization due to morbidity and social isolation (the latter perhaps impacting willingness or the ability to participate in a phone survey; the former impacting availability in the sampling frame). These factors may disproportionately weaken the adversity signal in older survey populations. However, demographic sensitivity analysis demonstrated that selective attrition alone cannot fully account for the cross-sectional differences observed in crude, age-specific ACE prevalence. For healthy survivor bias to be the sole explanation for a cross-sectional gradient (e.g., the ≈10-percentage-point difference in ≥4 ACE prevalence observed between young adulthood and older age in population surveys), high-ACE individuals would need a relative survival ratio of ${RR}_{surv}\approx 0.30$ to age 70 years. This would require an all-cause mortality hazard ratio $\left(HR>6.0\right)$ across adulthood that substantially exceeds empirical hazard ratios observed in prospective studies (*HR* = 1.3 to 1.8; Brown et al., 2009). Furthermore, while extreme exposure (≥6 ACEs) produces severe premature mortality (~20-year reduction in lifespan; Brown et al., 2009), this stratum represents <5% of the population, making it mathematically insufficient to account for broad cohort-level shifts. Thus, while selective attrition weakens the adversity signal among older cohorts—a dynamic formally modeled via our quadratic age specification—it operates alongside true cohort-level increases in exposure.

Our Age–Period–Cohort (APC) approach (Yang & Land, 2013) addresses these concerns by providing a survivor-adjusted estimation of cohort-specific ACE occurrence, allowing us to examine historical patterns despite missing data from earlier generations. To bridge these gaps, the APC model utilizes the overlapping experiences of successive cohorts captured at different periods. Specifically, by comparing the reporting patterns of a given age group across multiple survey years, we can estimate net cohort effects, which travel with that group across their lifespan, while accounting for age-specific mortality and morbidity. Under the specified identification assumptions, this approach allows us to generate estimated trajectories of adversity for earlier birth cohorts, helping to distinguish genuine historical change from artifacts of survivorship or selective recall.

## 2. Materials and Methods

### 2.1. Data Source

Data for this study were drawn from the BRFSS, a state-based surveillance system supported by the U.S. Centers for Disease Control and Prevention (CDC). A detailed description of the survey design and random sampling procedures is available elsewhere (www.cdc.gov/brfss; accessed 6 June 2026). Briefly, BRFSS collects data on many health-related risk behaviors and chronic conditions among U.S. adults (aged 18 and older). Trained interviewers collect data on a continuous, year-round basis using a dual-frame sampling design (landline and cellular telephones) to improve coverage of the non-institutionalized U.S. adult population; prior to 2011, only landline numbers were sampled. Data are weighted using iterative proportional fitting (raking) to improve population representativeness and reduce bias, a shift from a post-stratification approach used prior to 2011.

Since 2009, the BRFSS has offered an optional state module of questions about ACEs (Table 1). While every U.S. state has implemented this module at least once as of 2024, data availability is constrained by inconsistent participation and limitations of inclusion in the national public use data files. Specifically, of the identified 213 instances where states included the ACE module between 2009 and 2024, 120 were not included in the CDC’s public-use data files (PUF). Notably, this includes a complete absence of ACE module data in the national PUF during the years 2013–2018, despite some individual state-level collection during that period. Consequently, our dataset is a robust but specific subset of the total state-collected childhood adversity data.

To ensure consistency across the included survey years, the cumulative ACE score was restricted to the component ACEs captured by the ACE module (Table 1) that are present in each of the survey years of 2009–2012 and 2019–2024. These include three categories of abuse (physical, emotional, and sexual abuse) and five categories of household adversities (household member substance use, mental illness, incarceration, parental separation/divorce, and witnessing intimate partner violence). While more recent (2021–2024) BRFSS surveys have introduced a measure of neglect, this was excluded from the present analysis. All questions refer to the time period before the respondents were 18 years old. Each respondent’s ACE score was computed as the sum of the scores for the eight dichotomous, component ACEs, resulting in a value ranging from zero to eight. Respondents missing data for any of the ACE components were excluded from the primary analysis.

The primary aim of this analysis was not to produce a nationally representative point-prevalence of ACEs for any single year. Instead, our objective was to leverage the high volume of individual ACE module responses to analyze historical patterns in the prevalence of ACEs across birth cohorts while accounting for normative cultural trends in modern reporting. To achieve the sample density required for Age–Period–Cohort (APC) modeling described below, we pooled public-use datasets from two distinct periods that align with the availability of the ACE module described above within the CDC’s public-use files:

- The 2009 to 2012 surveys, which capture the initial years of ACE module implementation:
- The 2019 to 2024 surveys, which capture the most recent data inclusive of contemporary shifts in ACE disclosure.

By pooling these datasets, we created a large, synthetic cohort of the U.S. adult experience with childhood adversity (data were available for *n* = 517,296 observations for the primary analysis). While the underlying surveys are cross-sectional, the APC framework allows us to track the evolution of childhood adversity of birth cohorts across time, prioritizing historical signals over geographical representation and providing a unique window into how childhood has changed in the U.S. with regard to ACEs over the last 80 years.

In pooling BRFSS data across survey years, for each annual dataset, we preserved year-specific survey designs. This was accomplished by creating unique strata and primary sampling unit (PSU) identifiers based on an interaction of the survey year indicator with the original BRFSS design variables (strata and PSU). This ensured that strata and PSU codes were unique across years in the pooled file. In addition, we normalized analytic weights by computing the sum of the BRFSS final analysis weights among completed interviews within each annual dataset. Following integration of all annual datasets, normalized weights were rescaled to the pooled analytic sample size by multiplying by the total number of observations in the complete dataset. Consequently, our pooled results should be interpreted as design-adjusted patterns among respondents across years, not population-representative estimates for the U.S.

### 2.2. Univariate Analysis

We calculated the mean ACE score and corresponding 95% confidence intervals (CIs) for each individual birth cohort from 1925 to 2003. These estimates served as the basis for our historical trend analysis. The same process was used to obtain the prevalence of high ACE scores (ACE score ≥ 4) for each birth cohort from 1925 to 2003. To mitigate stochasticity and year-to-year volatility, particularly in birth cohorts with lower representation in the pooled BRFSS dataset, we applied a 5-year centered rolling average smoothing technique. We selected a five-year window whereby for any index year (*t*), the smoothed value was computed as the arithmetic mean of the raw estimates for years *t* − 2, *t* − 1, *t*, *t* + 1, and *t* + 2. This smoothing was applied consistently to the mean ACE scores and the lower and upper bounds of the 95% CIs. By utilizing this rolling average, we stabilized year-over-year volatility while maintaining the underlying longitudinal trend, ensuring that each cohort’s estimate remains anchored to the experience of adjacent birth years. The same process was used for calculating the prevalence of high ACE scores for each birth cohort.

After the initial smoothing, we grouped the data into five-year bins (e.g., 1925–1929, 1930–1934, 1995–1999, and 2000–2003) to facilitate longitudinal comparison. Each bin’s value was calculated as the arithmetic mean of the rolling averages within that specific timeframe. This two-step approach was used to stabilize the trend line and minimize the influence of annual volatility on cohort-specific results.

It is important to note a limitation regarding the beginning and tail-end of our time series. Because the birth years of our birth cohorts span 1924–2003 (persons aged 18 years at the time of the 2024 survey), the 5-year rolling windows for the first and final two years are incomplete (e.g., for 2003, data for the future years 2004 and 2005 were unavailable at the time of analysis). Consequently, the estimates for the first (1925–1929) and final bins (2000–2003) reflect this boundary constraint. While this results in a slight reduction in the smoothing power at the tails of the series, we believe the volume of data remains sufficient to identify the patterns noted in the results.

### 2.3. Multivariate Age–Period–Cohort Analysis

A fundamental identification problem exists in the analysis of variables involving Age (time since birth), Period (survey year), and Cohort (birth year) since they are conceptually perfectly collinear: Cohort = Period − Age. As such, we cannot include Age, Period, and Cohort simultaneously as independent variables in a standard linear regression. To address this problem, we employed a specific Age–Period–Cohort (APC) modeling strategy (Yang & Land, 2013) that allows us to distinguish these overlapping effects and model the mean ACE score for each person.

To address the inherent collinearity between Age, Period, and Cohort, we employed a constraint-based fixed effects approach and modeled the temporal components as follows:

- Age (Biological/Recall Effect): Modeled parametrically using a quadratic term (Age + Age^2^) based on the ordinal 5-year interval scale, capturing non-linearities in ACE reporting such as potential “recall fade” in older respondents or developmental differences in perception.
- Period (Cultural/Reporting Effect): Modeled using categorical fixed effects for each survey year with 2019 as the reference. The resulting IRRs for survey years measure shifts in disclosure behavior over time, likely linked to broader changes in social awareness and the reduction in stigma surrounding childhood adversity.
- Cohort (Historical/Generational Effect): Modeled as categorical fixed effects to identify unique historical shifts in childhood adversity exposure. By using cohorts born before 1930 as the baseline, we can interpret the IRRs as a measure of historical change. For example, an IRR of 1.25 suggests that a specific birth group experienced a 25% higher mean ACE burden than the reference cohort.

Thus, while Period and Cohort were modeled strictly as unconstrained categorical fixed effects (dummy variables), Age was modeled as a continuous parametric function rather than a set of independent categorical dummy variables. This asymmetry in functional form is what breaks the exact linear dependence among the three terms, permitting model identification and convergence. Our specification followed iterative testing, in which alternative models (including a fully categorical Age term) failed to converge. The appropriateness of the quadratic Age specification was supported by an adjusted Wald test, which confirmed that the Age^2^ term was a statistically significant contributor to model fit (F(1, 508,662) = 107.08, *p* < 0.001). We also compared the primary quadratic Age specification against a restricted linear-Age model using unweighted analyses; the Akaike Information Criterion (AIC) and Bayesian Information Criterion (BIC) both favored the quadratic specification.

Two distinct iterations of the model were implemented to ensure the stability of the generational signal:

- Model 1 (Single-Year Birth Cohort): This model utilized individual birth cohort years to provide a detailed view of historical risk.
- Model 2 (Binned Birth Cohort): To minimize year-to-year stochasticity and facilitate comparison with our univariate results, this model used birth years grouped into 5-year bins (e.g., 1935 to 1939, 1940 to 1944, etc.).

To examine patterns in mean ACE scores, a non-negative count ranging from zero to eight, we utilized a Poisson regression model to estimate Incidence Rate Ratios (IRRs), which represent the relative difference in the mean number of ACEs reported by a group compared to a baseline or reference. Poisson regression is standard for count outcomes of this nature and yields directly interpretable relative measures. To assess potential overdispersion, we fit a parallel survey-weighted negative binomial regression model. The estimated dispersion parameter (alpha = 0.679, 95% CI: 0.667–0.691) indicated statistically significant overdispersion relative to the Poisson equidispersion assumption. Despite this, comparisons of IRRs, CIs, and the pattern of statistical significance across all Age, Period, and Cohort terms were materially unchanged between the Poisson and negative binomial specifications, indicating that our substantive conclusions are robust to this distributional violation. Because our Poisson models were estimated using survey-design-based (linearized) standard errors, which do not rely on the Poisson mean–variance equality assumption, and because this sensitivity analysis found no material difference in results under the negative binomial alternative, overdispersion does not appear to have distorted our inferential conclusions. We therefore retained the Poisson specification—which remains statistically consistent for the conditional mean regardless of the true dispersion structure provided the mean model is correctly specified (Cameron & Trivedi, 2013)—due to its interpretability and consistency with our primary analytic approach.

We utilized Stata version 14 (StataCorp LLC, College Station, TX, USA) *svy* suite to respect the complex survey design of the BRFSS (stratification, clustering, and weighting) for all analyses, as it provides robust standard errors and unbiased point estimates. Given that multiple years of BRFSS data were pooled, standard regression techniques would likely underestimate the variance and produce artificially narrow confidence intervals. Our approach accounted for the sub-population of interest (respondents with complete ACE data) while maintaining the integrity of the original sampling weights, clusters (primary sampling units), and strata. Google Gemini (version 3 Flash) was used to assist with the drafting and refinement of Stata code for the Age–Period–Cohort models described above; all code was reviewed and validated by the authors.

## 3. Results

### 3.1. Univariate Analysis

**Figure 2:**
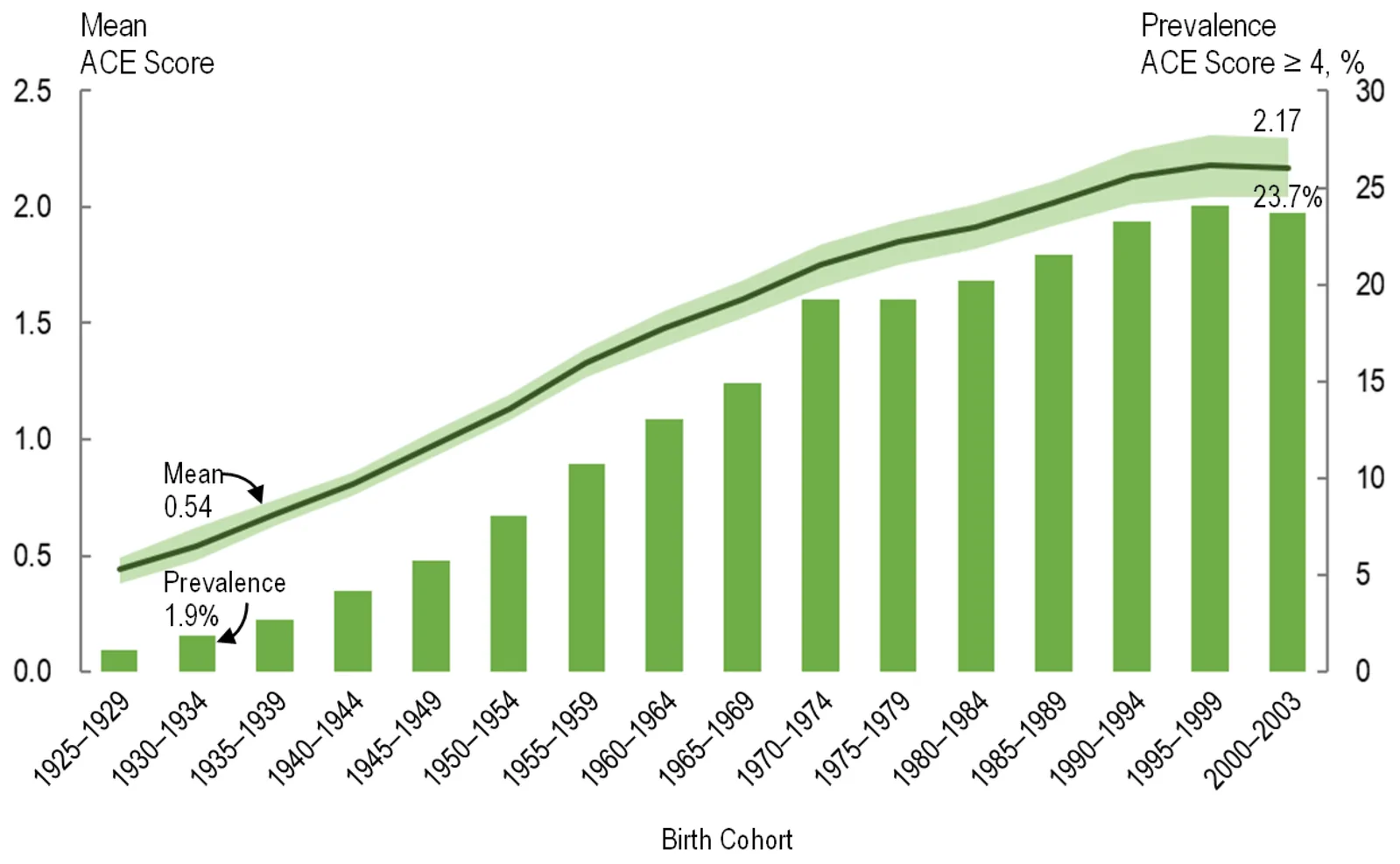
Five-year-binned summaries of smoothed average ACE scores and prevalence of high ACE scores (ACE score ≥ 4) by birth cohorts, 1925–2003.

We observed meaningful increases in the average ACE score per person and the prevalence of ACE scores ≥4 across successive birth cohorts from the 1930s through to the early 1990s, followed by a leveling through the early 2000s (Figure 2). Around 1930, ACEs were generally a rare event, with a mean ACE score of less than 1. As time moved forward, the mean ACE score and prevalence of high ACE scores increased for people born in the 1950s and 1960s, with a particular increase in the early 1970s. By the late 1990s and early 2000s, nearly one in four adults, particularly young adults at the time of survey, reported ≥4 ACEs.

We compared the mean ACE scores for adults aged 25–39 years during the 2009 and 2010 survey period (reflecting persons born around 1970–1985 or late Gen Xers/early Millennials) with those of similarly aged adults during the 2023 and 2024 survey period (reflecting persons born around 1984–1999 or late Millennials/Gen Zs). Holding age constant, we observed a meaningfully greater mean ACE score and prevalence of ACE score ≥4 for respondents in the 2023 and 2024 survey period (mean: 2.15, 95% CI: 2.09, 2.21; ≥4: 23.7%, 95% CI: 22.4, 25.0) compared to respondents in the 2009 and 2010 survey period (mean: 1.69, 95% CI: 1.59, 1.78; ≥4: 15.8%, 95% CI: 14.0, 17.7), which suggests a shift (27% increase in mean ACE score for the same stage in life) in the underlying childhood adversity profile over a 10–15 year period.

### 3.2. Trends in ACE Score Components

**Figure 3:**
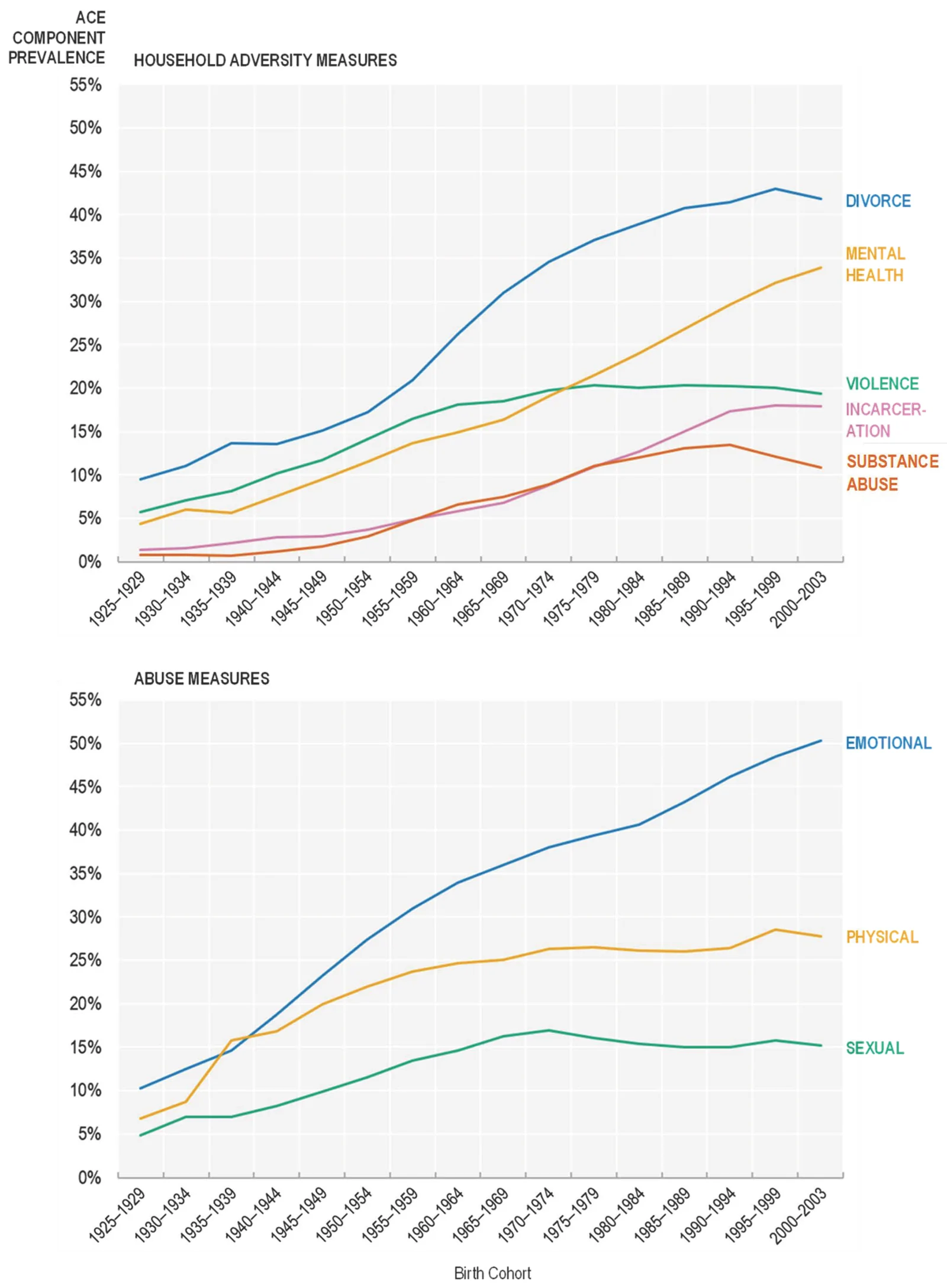
Summary of the prevalence of individual ACEs across birth cohorts.

The observed escalation in cumulative ACE scores across birth cohorts (Figure 2) is characterized by non-uniform trajectories among the individual components of the ACE score (Figure 3). The most pronounced increases were observed in household adversities linked to shifting social and structural landscapes. Specifically, the prevalence of an incarcerated household member rose from 1.4% (95% CI: 0.9, 2.2) in the 1925–1929 cohort to 17.9% (95% CI: 15.6, 20.5) in the 2000–2003 cohort, while parental divorce or separation quadrupled from 9.5% (95% CI: 7.8, 11.6) to 41.8% (95% CI: 38.7, 45.0) over the same period.

In contrast, reports of physical abuse, sexual abuse, and intimate partner violence exhibited a period of escalation mid-century followed by relative stabilization beginning in the 1970s. For example, reported sexual abuse prevalence reached 16.9% (95% CI: 15.2, 18.8) in the 1970–1974 cohort and remained statistically unchanged at 15.2% (95% CI: 12.9, 17.8) in the 2000–2003 cohort. Conversely, emotional abuse and household mental illness demonstrated sustained upward trajectories through the most recent cohorts, with emotional abuse surpassing 50% prevalence in the 2000–2003 cohort. These component-level shifts suggest that the stabilization of the cumulative ACE score (both the mean and prevalence of score ≥4) in recent years is the result of a compositional transition in the nature of reported household adversity.

### 3.3. Multivariate Age–Period–Cohort Analysis

**Figure 4:**
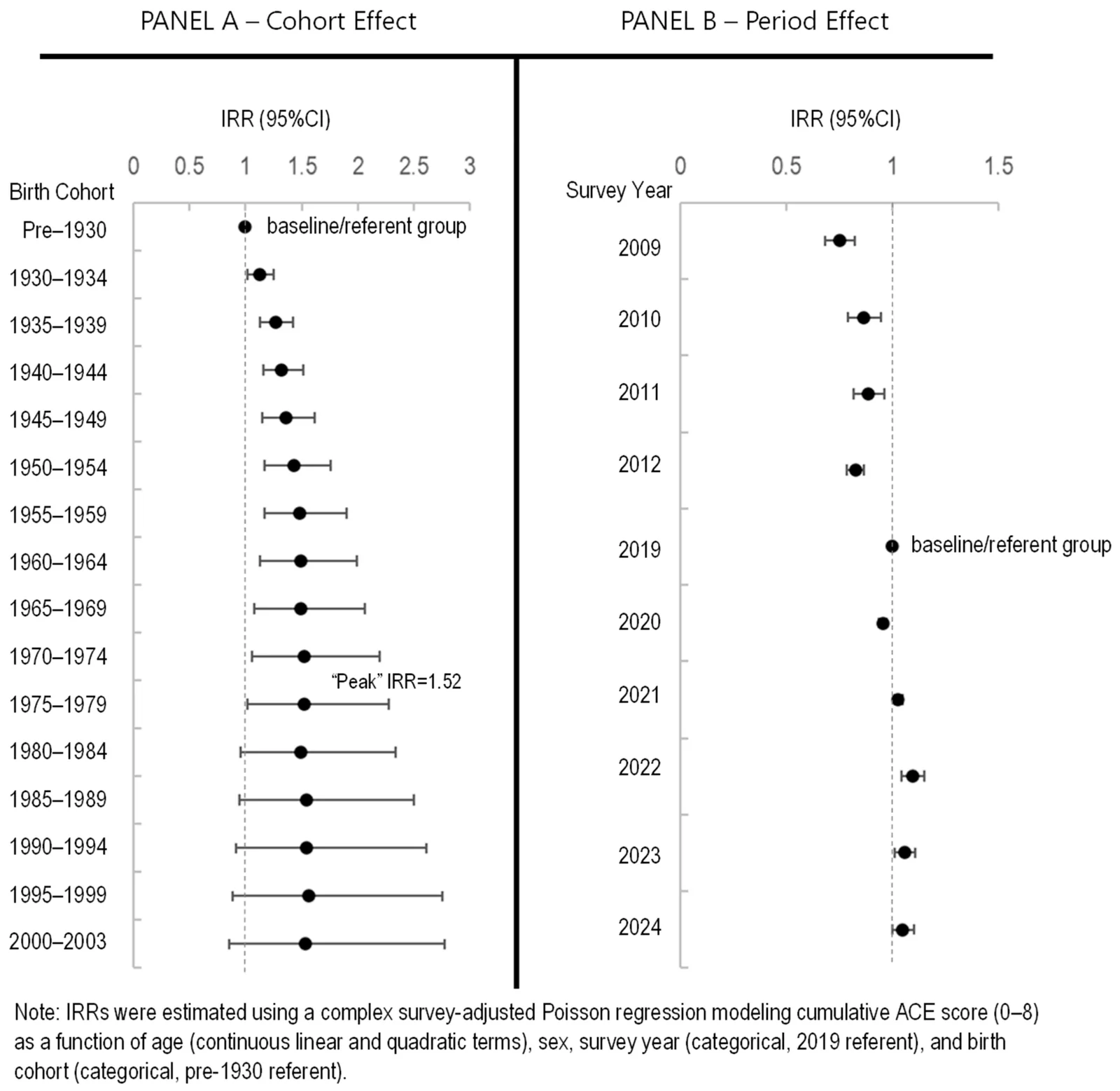
Summary of cohort and period effects on the expected mean ACE score, estimated using incidence rate ratios (IRRs).

Panel A of Figure 4 shows an estimate of the historical trend or signal based on when respondents were born (i.e., birth cohort effects) under the specified APC identification assumptions. By controlling for Period effects (the fact that we are more aware of the components of childhood adversity in 2024 than we were in 2010) and Age effects (the influence of memory, evolving perceptions, and the selective censoring of the population due to ACE-related morbidity and premature mortality), we were able to observe the impact of the era into which a person was born, the Cohort effect. The results reveal an increase in adversity that began with the cohorts of the late 1930s and reached a statistically significant peak among individuals born in the late 1950s, 1960s, and 1970s. These persons have a roughly 50% higher expected mean ACE score (IRR: 1.49 (1955–1959); 1.50 (1960–1964); 1.49 (1965–1969); 1.52 (1970–1974); 1.52 (1975–1979)) compared to those born before 1930. This divergence persists even when comparing individuals at the same age and within the same survey period, indicating that the shift is a reflection of historical occurrence rather than merely a change in retrospective interpretation.

We observed that point estimates for cohorts born during the 1980s, 1990s, and early 2000s remained roughly 50% higher than the baseline (reaching an IRR of 1.56 for the 1995–1999 cohort), but they were no longer statistically significant (*p* > 0.05). Even though the results for the younger cohorts were no longer statistically significant in this specific model, the fact that their point estimates remained elevated suggests that the high-risk environment of the 1950s, 1960s, and 1970s did not disappear. Rather, because these individuals are younger, they only appear in the most recent Period blocks of our data (2019–2024), and therefore, we have a narrower observation window for them, which creates greater statistical noise and wider confidence intervals in the results.

While Panel A of Figure 4 reveals a historical rise in childhood adversity *occurrence* over time, Panel B shows the estimated temporal shifts in the *reporting* of ACEs. By adjusting for age and birth cohort, the model quantifies changes in reported ACE counts attributable solely to the year of survey administration. Using 2019 as the baseline reference year (IRR = 1.0), the results indicate a statistically significant change in reporting norms over the 15-year period. The expected mean ACE counts for respondents in 2009 were nearly 25% less (IRR = 0.75) than those for respondents in 2019. In contrast, the expected mean ACE count for a demographically equivalent individual surveyed in 2022 was 10% higher than for respondents in 2019 (IRR = 1.10). Although estimated ACE burden declined slightly in 2020 (IRR = 0.95)—coinciding with the acute societal disruptions of the COVID-19 pandemic—the IRR remained consistently above the 2019 reference from 2021 to 2024. These findings suggest that enhanced public discourse surrounding childhood adversity, mental health, and systemic inequities has fostered a sustained environment of increased disclosure propensity.

Finally, the model demonstrates a significant gender-based disparity, with females reporting a 16% higher expected mean ACE score than males (IRR = 1.16; 95% CI: 1.15, 1.18; *p* < 0.001) after adjusting for birth cohort and survey year. Furthermore, the quadratic age term yielded an IRR of 0.992 (95% CI: 0.991, 0.994; *p* < 0.001), identifying a subtle non-linear association where the expected ACE count declines marginally as age increases. This trend is consistent with retrospective recall attenuation or gradual decay of childhood memories over the life course and healthy survivor bias, which was noted at the outset, whereby individuals with high cumulative childhood adversity burdens are likely underrepresented in older age groups due to premature mortality.

## 4. Discussion

As noted in the Introduction, a central methodological concern for this analysis is whether “healthy survivor bias”, or the selective loss of high-adversity individuals from older cohorts through premature mortality or institutionalization, could alone explain the generational pattern we observed. For this to be the sole explanation, ACE-related mortality and non-response would have to occur among earlier cohorts at levels that exceed what is documented in population vital statistics. Our findings indicate that the transition observed cannot be attributed solely to differential mortality or evolving reporting norms. Rather, the evidence points to a genuine historical increase in childhood adversity across successive generations, occurring alongside a period effect likely reflecting a simultaneous shift in disclosure norms.

The findings of this study signal an important demographic shift: a transition from a society where the prevalence of childhood adversity was a marginal public health or societal concern to a contemporary reality where ACEs are a consequential public health problem. This is most vividly illustrated by the shifting profile of young, parenting-age adults. In just over a decade, the mean ACE score for this demographic has risen from 1.7 in 2010 to 2.2 in 2024. While our Age–Period–Cohort model suggests that the historical occurrence of adversity has reached a high-risk plateau (where nearly 25% of young adults report four or more ACEs), this stabilization at an elevated level must not be mistaken for a reversal of trends. Rather, the structural conditions that drove the trend in reported ACEs in the mid-to-late 20th century appear to have resulted in a sustained high-prevalence stabilization. For the young adults of today, the high prevalence of childhood adversity is no longer an escalating risk but an entrenched baseline reality. Concurrently, we observed a significant Period effect: the expected mean ACE score varied meaningfully across survey years, independent of birth cohort or age. We hypothesize that this reflects modern normative changes in the identification, discussion, and reporting of these experiences, including a proliferation of “trauma-informed” language in schools and a broad destigmatization of mental health—as our cross-sectional data cannot directly measure disclosure behavior or perceptions, this interpretation would benefit from confirmation using measures of awareness or disclosure attitudes in future research. This suggests that a 30-year-old today is not only navigating the consequences of a historically riskier childhood landscape but is also operating within a cultural environment characterized by greater awareness and discussion of ACEs.

Let us put this into a different context. Imagine three people receiving services today: a woman born in the 1940s, who is now in her late 70s to early 80s; a parent born in the 1970s, who is now in her late 40s to early 50s; and a young woman born in the early 2000s, who is now in her early 20s. For the oldest woman, a high-ACE childhood was uncommon; only about 1 in 20 of their peers experienced ACE scores ≥4. Our middle-aged parent, however, experienced a massive shift. By the time they were growing up, nearly one-in-five of her peers were experiencing high ACE levels. And finally, for our young adult representing the next generation of parents, high levels of childhood adversity are a common reality, as one in four of her peers report a high ACE score.

The observed gender-based disparity, with females reporting higher expected mean ACE scores than males, is consistent with prior population-based research showing that women report higher cumulative ACE scores than men, a difference driven substantially by higher reported rates of childhood sexual abuse and, to a lesser extent, household mental illness or substance use exposure, rather than a uniform elevation across all ACE categories (Dube et al., 2005). This suggests that the gender disparity in our data likely reflects differential exposure to specific, well-documented forms of adversity rather than a general reporting artifact, though we cannot rule out differential willingness to disclose certain experiences between genders in our cross-sectional design.

It is worthwhile to consider patterns observed in Figure 2, including the early rise in ACEs and what may explain the large increase in prevalence of high ACE scores. The rise in the expected mean ACE score beginning with cohorts born in the early-to-mid 20th century may in part reflect the economic and social disruption of the Great Depression, which is documented to have strained family stability and marital relationships during this period (Elder, 1999). We note this as a plausible historical contributor rather than a tested mechanism; our cross-sectional, retrospective data cannot establish a causal link between Depression-era conditions and the cohort-level ACE signal we observe, and we did not identify strong empirical evidence for a comparable effect of World War II-era family disruption specifically. For those born in the late 1960s and early 1970s (Gen X), sociology and history offer several reasons as to *why* childhood became more turbulent for this specific group. The “Divorce Revolution” between the 1960s and 1980s saw the U.S. divorce rate more than double, making parental separation a normative experience for the first time (Stevenson & Wolfers, 2007). Simultaneously, the “war on drugs” and a shift in sentencing laws catalyzed an increase in the U.S. prison population, directly increasing the prevalence of having an incarcerated household member (Wildeman, 2009). The substantial economic stress of the 1970s created a backdrop of parental economic shocks, which is a known catalyst for household stress, substance use, and mental health struggles (Elder, 1999). Furthermore, the passage of the Child Abuse Prevention and Treatment Act (CAPTA) in 1974 (Child Abuse Prevention and Treatment Act, 1974) initiated the era of mandatory reporting; while this did not necessarily create more abuse or prevent it, CAPTA institutionalized the process of identifying and naming it. This historical hypothesis is supported by the component-level data, which shows that the prevalence of parental divorce and household incarceration did not merely rise, but fundamentally transformed the childhood experience for the 1970s’ birth cohorts and beyond.

The stabilization of a high ACE prevalence and estimated expected mean ACE scores in the most recent cohorts must not be mistaken as a reversal of intergenerational trends. Informed by the literature on the intergenerational transmission of stress (Yehuda & Lehrner, 2018; Conching & Thayer, 2019), it has been hypothesized that cohorts born between 1980 and 2000 may function as a “bridge generation”, where the delayed or latent expression of earlier ancestral or historical exposures could influence subsequent generations. We emphasize, however, that our cross-sectional surveillance data cannot test intergenerational transmission or epigenetic mechanisms directly. This concept is presented purely as a speculative framework to guide future prospective research rather than as a conclusion drawn from our models.

### 4.1. A Structural Mismatch: Legacy Systems in a High-Prevalence Society

A societal transition characterized by a shift from a low prevalence to a high prevalence of childhood adversity carries profound implications for the American social safety-net and publicly funded systems (e.g., education, health, justice, social welfare, etc.). The bulk of our foundational social safety-net systems (e.g., social welfare/AFDC, Medicaid, the Food Stamp Act, and Head Start) were developed in the mid-20th century when ACE scores ≥4 were far less common and not yet a recognized feature of the broader American demographic landscape. These systems reflect mid-century policy-making that prioritized isolated, single-sector interventions rather than integrated care, built without today’s understanding of the health and social impacts of childhood adversity. The life course impact of ACEs, however, is not episodic but rather cumulative and wide-ranging (Anda et al., 2006), shaping trajectories in educational attainment (Metzler et al., 2017; Blodgett & Lanigan, 2018), employment (Hardcastle et al., 2018), health (Anda et al., 2006), social relationships (Curtis et al., 2025), and community participation (Klein et al., 2025). Yet current responses remain siloed: health, education, child welfare, and justice systems each address the consequences of adversity within their own domain and at specific intervals, rather than through coordinated, cross-sector strategies that emphasize primary, secondary, and tertiary prevention and reducing downstream risk and progression. Much like an emergency department locked in a cycle of acute triage, these systems are structured to treat the immediate presenting crisis, without the capacity to address the underlying, highly endemic adversity driving the caseload (Halfon & Hochstein, 2002). By treating each outcome as a separate service issue, we fail to address the cumulative, cross-sector burden that our model identifies. If we continue to ignore this mismatch between system design and population need, we risk failing the bridge generation and the children of the next generation.

### 4.2. Assumptions and Limitations

There are several assumptions and limitations to note. First, our APC specification required a constraint-based identification strategy (detailed in Section 2) to break the exact collinearity among Age, Period, and Cohort. While our chosen constraint was empirically supported and alternative reference cohorts produced a consistent trend, the APC models remain inherently sensitive to the identifying assumptions selected, and a differently specified model—for example, one that constrains Cohort rather than Age—could in principle yield different absolute IRR magnitudes, even if the broader historical signal proved robust across the specifications we tested. Second, we tested multiple reference cohorts to ensure the robustness of the historical signal. While the absolute magnitude of the IRRs is sensitive to the choice of the reference—with the pre-1930 anchor revealing a more pronounced escalation than more recent baselines—the relative direction and shape of the trend remained consistent across all specifications. Third, our use of Poisson regression assumes equidispersion; a negative binomial sensitivity analysis (detailed in Section 2) confirmed that our substantive conclusions were unchanged under this alternative distributional assumption, but this does not establish that the Poisson mean–variance assumption itself holds, only that our results are not sensitive to relaxing it. Finally, our analysis was subject to sampling constraints. Because the BRFSS only interviews non-institutionalized adults aged 18 years or older, and the 2024 survey was the most recent available survey at the time of analysis, the most recent cohorts (post-2003) were not fully represented. Our analysis was therefore truncated at the 2003 birth cohort. Furthermore, as a survey of non-institutionalized adults, these data may underestimate ACE prevalence among populations experiencing higher rates of incarceration or residential treatment, who often carry disproportionately high exposure to child adversity. In addition, although many states were not represented because the ACE module was not used in a BRFSS, we did not observe meaningful differences in background demographics between the respondents included in the primary analysis and those that were not.

## 5. Conclusions

This analysis delineates a fundamental transformation in the American childhood landscape. By analyzing the estimated cohort and period trends derived from our Age–Period–Cohort modeling framework, we observed several patterns that are important for future public health and social policy. First, we observed a shift from a society characterized by low prevalence of ACEs to one where high prevalence of childhood adversity is the norm. The 50% increase in the expected mean ACE score among mid-century cohorts relative to the pre-1930 baseline represents a structural shift that has since stabilized as a high-risk plateau among today’s young adults. Second, the most recent surge in reported ACE scores is driven by a powerful Period effect, likely reflecting a shift toward greater disclosure of ACEs, potentially linked to destigmatization of mental health issues and abuse reporting, an increase in trauma-informed service delivery, and a broader cultural vocabulary for childhood adversity compared to previous decades. Third, while the ACE scores of more recent cohorts appear to have stabilized at a high plateau, whether these birth groups carry underlying biological or social vulnerabilities that affect subsequent generations remains an open, untested question. Because our cross-sectional design cannot evaluate intergenerational transmission, dedicated longitudinal and biomarker studies will be required to investigate these broader generational dynamics.

Addressing this shift will require confronting the structural mismatch between a 21st-century, high-ACE-prevalence reality and legacy social safety-net systems designed for discrete, episodic needs in the mid-20th century. Moving beyond a reactive, siloed service model toward integrated, intersectoral approaches—spanning healthcare, education, social services, justice, and housing—is necessary to prevent a compounding intergenerational escalation of these trends.

## Figures and Tables

**Table 1:** Components, questions, responses, and scoring of the BRFSS ACE module.

ACE Component	Survey Question	Response Options	Scoring
**Childhood abuse**			
Physical abuse	How often did your parent or an adult in your home ever hit, beat, kick, or physically hurt you in any way? Do not include spanking.	Never/Once/More Than Once	1 = Once or More Than Once 0 = Never
Sexual abuse	How often did anyone at least 5 years older than you or an adult … … ever touch you sexually? … try to make you touch them sexually? … force you to have sex?	Never/Once/More Than Once	1 = Once or More Than Once to at least one of the three questions included in this category 0 = Never to all three questions in this category
Emotional abuse	How often did a parent or adult in your home ever swear at you, insult you, or put you down?	Never/Once/More Than Once	1 = Once or More Than Once 0 = Never
**Household Adversities**			
Mentally ill household member	Did you live with anyone who was depressed, mentally ill, or suicidal?	Yes/No	1 = Yes 0 = No
Substance abuse in household	Did you live with anyone who … … was a problem drinker or alcoholic? … abused prescription medications?	Yes/No	1 = Yes to one or both of the two category questions 0 = No to both questions
Incarcerated household member	Did you live with anyone who served time or was sentenced to serve time in a prison, jail, or other correctional facility?	Yes/No	1 = Yes 0 = No
Violence between adults in household	How often did your parents or adults in your home ever slap, hit, kick, punch, or beat each other up?	Never/Once/More Than Once	1 = Once or More Than Once 0 = Never
Parental separation/divorce	Were your parents separated or divorced?	Yes/No	1 = Yes 0 = No

All questions refer to the time period before the respondents were 18 years old.

## Data Availability

All data used for this analysis are publicly available for download from www.cdc.gov/brfss.
